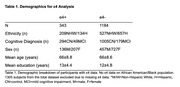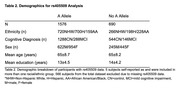# *APOE* and Brain Amyloid and Tau Levels

**DOI:** 10.1002/alz.091505

**Published:** 2025-01-09

**Authors:** Danielle Luu, Koral V Wheeler, Noelle Lee, Maxwell W Hand, Robert Barber, Nicole Phillips, Arthur W. Toga, Sid E. O'Bryant, Kristine Yaffe, Meredith N Braskie

**Affiliations:** ^1^ Imaging Genetics Center, Mark and Mary Stevens Neuroimaging and Informatics Institute, Keck School of Medicine, University of Southern California, Marina del Rey, CA USA; ^2^ Stevens Neuroimaging and Informatics Institute, Los Angeles, CA USA; ^3^ Stevens Neuroimaging and Informatics Institute, Marina del Rey, CA USA; ^4^ University of North Texas Health Science Center, Fort Worth, TX USA; ^5^ University of California, San Francisco, Weill Institute for Neurosciences, San Francisco, CA USA

## Abstract

**Background:**

The ɛ4 allele and promoter region variant, rs405509, of *APOE* are risk factors for late onset Alzheimer’s Disease (LOAD) (Raulin et al., 2022, Logue et al., 2023). Limited research exists on how these alleles affect LOAD pathology across ethno‐racial groups. We related risk allele dosage to brain amyloid and tau positron emission tomography (PET) signal.

**Method:**

We examined 2832 individuals (574 mild cognitive impaired, 2258 cognitively intact, mean age=65±8.5 years) from the Health and Aging Brain Study‐Health Disparities. This cohort includes self‐reported ethnoracial data including Non‐Hispanic White (NHW), Hispanic (H), and African American/Black (AA). Tau PET scans (PI 2620) had the medial temporal lobe (MTL), posterior cingulate (PC), and lateral parietal (LP) as ROIs and the inferior gray matter of the cerebellum as the reference region. We used amyloid (florbetaben) PET scans and calculated global amyloid as a mean of standardized uptake value ratio (SUVR) in frontal, cingulate, lateral parietal, and lateral temporal lobes with the whole cerebellum as the reference region. We ran linear regressions between risk allele dosage and amyloid/tau PET levels in the overall sample and stratified by race/ethnicity. Covariates included age, sex, education level, cognitive diagnosis, time between clinical visit and scan, and first two components from principal component analysis. We covaried for ɛ4‐positivity when available (Table 1) and scanner and corrected for multiple comparisons using Bonferroni correction.

**Result:**

In the total sample, more ɛ4 alleles were associated with more tau in the MTL (β= 0.38, p= 5.24e‐07) and LP (β= 0.26, p= 7.28e‐04). In NHW participants alone, this association persisted in the MTL (β= 0.38, p= 6.51e‐06) and LP (β= 0.31, p= 4.94e‐04), although without a significant race/ethnicity interaction. Amyloid and tau weren’t associated with rs405509 genotype in the overall sample, in subsets with/without an ɛ4 allele, or in ethnoracial subsets.

**Conclusion:**

There was a positive relationship between ɛ4 dosage and tau in the MTL and LP, particularly in NHW participants. There were no significant findings of rs405509 on amyloid/tau, or of either allele on tau in the PC.